# Mortality due to noncommunicable diseases in Brazil, 1990 to 2015, according to estimates from the Global Burden of Disease study: Mortalidade por doenças não transmissíveis no Brasil, 1990 a 2015, segundo estimativas do estudo de Carga Global de Doenças

**DOI:** 10.1590/1516-3180.2016.0330050117

**Published:** 2017-04-03

**Authors:** Deborah Carvalho Malta, Elisabeth França, Daisy Maria Xavier Abreu, Rosângela Durso Perillo, Maíra Coube Salmen, Renato Azeredo Teixeira, Valeria Passos, Maria de Fátima Marinho Souza, Meghan Mooney, Mohsen Naghavi

**Affiliations:** I MD, PhD. Professor and Researcher, Department of Mother and Child and Public Health, Nursing School, Universidade Federal de Minas Gerais (UFMG), Belo Horizonte (MG), Brazil.; II MD, PhD. Associate Professor, Department of Preventive and Social Medicine, Universidade Federal de Minas Gerais (UFMG), Belo Horizonte (MG), Brazil.; III PhD. Researcher, Nucleus of Education in Collective Health, School of Medicine, Universidade Federal de Minas Gerais (UFMG), Belo Horizonte (MG), Brazil.; IV MSc. Nurse, Municipal Health Department, Belo Horizonte, and Researcher, School of Medical Sciences, Universidade Federal de Minas Gerais (UFMG), Belo Horizonte (MG), Brazil.; V BA. Researcher and Strategic Planning Manager, Hospital Israelita Albert Einstein (HIAE), São Paulo (SP), Brazil.; VI Statistician, Postgraduate Program on Epidemiology, Faculdade de Medicina, Universidade Federal de Minas Gerais (UFMG), Belo Horizonte (MG), Brazil.; VII MD, PhD. Professor, Faculdade de Ciências Médicas de Minas Gerais, Belo Horizonte (MG), Brazil.; VIII MD, PhD. Director, Ministry of Health, Brasília (DF), Brazil.; IX PhD. Senior Engagement Manager in the Global Engagement Team, Institute for Health Metrics and Evaluation (IHME), Washington DC, United States.; X MD, MPH, PhD. Professor of Global Health, Institute for Health Metrics and Evaluation (IHME), Washington DC, United States.

**Keywords:** Chronic disease, Neoplasms, Diabetes mellitus, Respiratory tract diseases, Global burden of disease

## Abstract

**CONTEXT AND OBJECTIVE:**

Noncommunicable diseases (NCDs) are the leading health problem globally and generate high numbers of premature deaths and loss of quality of life. The aim here was to describe the major groups of causes of death due to NCDs and the ranking of the leading causes of premature death between 1990 and 2015, according to the Global Burden of Disease (GBD) 2015 study estimates for Brazil.

**DESIGN AND SETTING:**

Cross-sectional study covering Brazil and its 27 federal states.

**METHODS:**

This was a descriptive study on rates of mortality due to NCDs, with corrections for garbage codes and underreporting of deaths.

**RESULTS:**

This study shows the epidemiological transition in Brazil between 1990 and 2015, with increasing proportional mortality due to NCDs, followed by violence, and decreasing mortality due to communicable, maternal and neonatal causes within the global burden of diseases. NCDs had the highest mortality rates over the whole period, but with reductions in cardiovascular diseases, chronic respiratory diseases and cancer. Diabetes increased over this period. NCDs were the leading causes of premature death (30 to 69 years): ischemic heart diseases and cerebrovascular diseases, followed by interpersonal violence, traffic injuries and HIV/AIDS.

**CONCLUSION:**

The decline in mortality due to NCDs confirms that improvements in disease control have been achieved in Brazil. Nonetheless, the high mortality due to violence is a warning sign. Through maintaining the current decline in NCDs, Brazil should meet the target of 25% reduction proposed by the World Health Organization by 2025.

## RESUMO

**CONTEXTO E OBJETIVO:**

As doenças crônicas não transmissíveis (DCNT) são o principal problema de saúde global e geram um elevado número de mortes prematuras e perda de qualidade de vida. O objetivo foi descrever os principais grupos de causas de morte por DCNT e o *ranking* das causas de morte prematura entre 1990 a 2015, segundo estimativas do estudo Global Burden of Disease (GBD) 2015 para o Brasil.

**TIPO DE ESTUDO E LOCAL:**

Estudo transversal do Brasil e 27 Unidades Federadas.

**MÉTODOS:**

Estudo descritivo das taxas de mortalidade por DCNT, com correções para sub-registro e códigos *garbage*.

**RESULTADOS:**

O estudo aponta a transição epidemiológica no Brasil entre 1990 e 2015, com o crescimento da mortalidade proporcional por DCNT, seguida das violências, e com a redução das causas maternas, infecciosas e infantis na carga global de doenças. As DCNT cursaram com as taxas de mortalidade mais elevadas em todo o período, mas com declínio para as doenças cardiovasculares, respiratórias crônicas e câncer. O diabetes aumentou no período. As DCNT lideram entre as causas de morte prematura (30 a 69 anos): doenças isquêmicas do coração e doenças cerebrovasculares, seguidas de violência interpessoal, lesão no trânsito e HIV/aids.

**CONCLUSÕES:**

A queda da mortalidade por DCNT confirma melhora do controle de doenças no país. Entretanto, a alta mortalidade por violência é um sinal de alerta. Mantendo-se a queda atual das DCNT, o Brasil deverá atingir as metas de redução propostas pela Organização Mundial de Saúde de 25% até 2025.

## INTRODUCTION

Noncommunicable diseases (NCDs) are the leading health problem globally and generate high numbers of premature deaths and loss of quality of life, thus imposing a high degree of limitation and disability, as well as economic impacts for families and society in general.^1^ The World Health Organization (WHO) has estimated that NCDs account for about 70% of all global deaths, i.e. about 38 million deaths annually. Among these deaths, 16 million occur prematurely (under 70 years of age) and almost 28 million are in low and middle-income countries.^2^ The NCD burden encompasses individuals in all socioeconomic strata and, more intensely, those belonging to vulnerable groups, such as the elderly and those with low levels of education and income.^3^

In Brazil, NCDs also constitute a major health problem and, according to data from the national mortality information system, they corresponded to 72.6% of the causes of death in 2011. Cardiovascular diseases ranked first among the causes of death, followed by neoplasms, chronic respiratory diseases and diabetes.^4^^,^^5^

There is strong evidence correlating social determinants such as education, occupation, income, gender and ethnicity with the prevalence of NCDs and risk factors such as tobacco use, harmful alcohol use, physical inactivity and unhealthy diet.^1^ If not properly prevented and managed, these diseases will require medical care with increasing costs due to incorporation of technology, in addition to causing loss of quality of life and premature deaths and having a great impact on the economy, thereby affecting countries’ growth and having the capacity to reduce gross domestic product (GDP) by up to 2% a year.^2^

In 2011, the United Nations (UN) recognized the magnitude of NCDs worldwide and established commitments to address this problem (United Nations, 2011) and, in 2013, WHO approved a global action plan for prevention and control of these diseases.^6^ This global plan defined a priority goal consisting of a 25% relative reduction in premature mortality due to NCDs (cardiovascular diseases, cancer, diabetes or acute respiratory diseases) among people between ages of 30 and 70 years by 2025.^6^ This overarching goal is consonant with the national target defined by the National Plan to Combat Noncommunicable Diseases 2011-2022.^7^

In 2015, the UN Assembly adopted a set of 17 goals as part of a sustainable development agenda, including the goal of “ensuring healthy lives and promoting well-being for all at all ages”. One of the targets was to “reduce by one third premature mortality due to non-communicable diseases through prevention and treatment and to promote mental health and wellbeing by 2030”, which continued the commitment already made by the World Health Assembly to achieve this by 2025.^8^

It has therefore become a global priority to monitor trends in mortality due to NCDs in order to ascertain whether national and global commitments will be achieved. Since 1990, the concept of the Global Burden of Disease (GBD) has grown in importance for monitoring the burden of disease in various countries. By 2015 all databases had been updated, thus making it possible to analyze information from 1990 to 2015 for most of the world’s countries, including Brazil and its 27 federal units (i.e. states), regarding premature death and disability due to more than 290 diseases, injuries and sequelae, along with the attributable burden due to risk factors, for 20 age groups and both sexes.^9^

The methodology used for the GBD consists of major advances and a shift in paradigm in epidemiological analysis on databases. An integrated approach towards diseases and deaths is proposed, with standard methodology for analysis and correction of underrreporting of deaths and garbage code. In this manner, it becomes possible to compare countries, regions and subnational data.^5^^,^^9^ The GBD study method enables users to assess burden trends, since time series data are adjusted and comparable.^5^^,^^9^

Thus, use of GBD estimates provides a great opportunity to move forward in assessing mortality due to NCDs, thereby supporting monitoring efforts and achievement of future global and national NCD reduction targets.^6^

## OBJECTIVE

The objective of this study was to describe the major groups of causes of death due to noncommunicable diseases (NCDs) and the ranking of the leading causes of premature death between 1990 and 2015, according to the GBD 2015 study estimates for Brazil.

## METHODS

This was a cross-sectional study based on data from the GBD 2015 study and also on the methods that it used, which have already been described in detail in another article.^9^

The GBD 2015 study used data on causes of death available from 195 countries, including Brazil and its 27 states. Information on causes of death was gathered from vital registration systems, mortality surveillance systems, surveys, hospital records, police records and verbal autopsies. The GBD uses methodologies for correcting underreporting of deaths and garbage code. Correction of the codes uses evidence from the medical literature, expert opinions and statistical techniques to designate each item to the most probable causes of death.^9^ In Brazil and its 27 federal states, the source of mortality data is the Mortality Information System (Sistema de Informação Sobre Mortalidade, SIM).^5^

After addressing data quality issues, the GBD 2015 study used a variety of statistical models to determine the number of deaths from each cause, through the cause of death ensemble model (CODEm) algorithm. To ensure that the number of deaths per cause did not exceed the total number of estimated deaths, a correction technique called CoCorrect was used. This technique makes certain that estimates of the number of deaths from each cause do not add up to more than 100% of deaths in a given year.^9^^,^^10^^,^^11^

After producing estimates for the number of deaths from each of the 249 fatal outcomes included in the list of causes of the GBD 2015 study, the years of life lost (YLLs) due to premature death were calculated. For every death due to a particular cause, the number of years lost was estimated based on the highest life expectancy in the deceased individual’s age group.^9^^,^^11^

The GBD study used a cause list that placed 249 causes of death within a four-level hierarchy. The first level divided causes into three groups: communicable, maternal, neonatal and nutritional conditions; NCDs and injuries. The second level consisted of 20 major causes of diseases such as neonatal disorders, cardiovascular diseases and traffic injuries. The third level subdivided level 2 into types such as neonatal preterm birth complications, cerebrovascular disease and traffic injuries; and the fourth level further subdivided those types in some cases, for example: ischemic stroke and hemorrhagic stroke; and pedestrian road injuries, cyclist road injuries, motorcyclist road injuries, motor vehicle road injuries and other road injuries.^9^

In the present study, the following NCDs were selected: cardiovascular diseases (I00-I99), respiratory diseases (J30-J98), neoplasms (C00-C97) and diabetes mellitus (E10-E14). The study used the concept of premature mortality, as previously used by WHO and the UN, respectively in the Global Plan for NCDs (2013) and in the Sustainable Development Goals (SDG) for NCDs (2015), which take premature deaths to be those occurring before the age of 70 years. The 20 leading causes of death were analyzed using the level 3 aggregation of causes of death from the GBD 2016 study, in the age group from 30 to 69 years. The results were compared between the years 1990, 2005 and 2015.

There was no need to submit this study to a research ethics committee, since it was conducted on a public-domain secondary database, without nominal identification, in accordance with Decree No. 7,724 of May 16, 2012, and Resolution No. 510 of April 7, 2016.

## RESULTS

**Figure 1:**
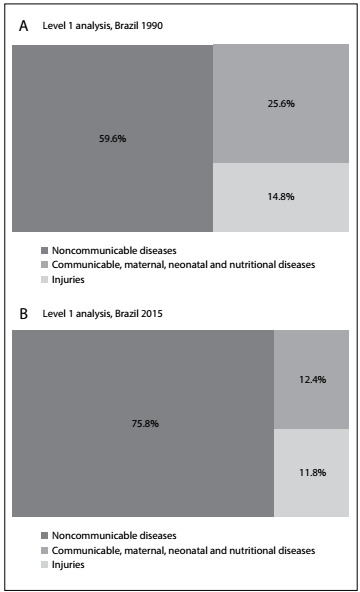
Proportions of numbers of deaths according to causes of death.

Figure 1 shows the relative distribution of deaths according to the three major groups of causes of death in Brazil for both sexes and the whole population. In 1990, proportional mortality due to NCDs corresponded to 59.6% of deaths (95% uncertainty interval, UI: 58.97-60.7); injuries, 14.8% (95% UI: 14.5-15.12); and communicable, maternal, neonatal and nutritional diseases, 25.6% (95% UI: 24.49-26.29). In 2015, proportional mortality due to NCDs had increased to 75.8% (95% UI: 75.05-77.30); injury deaths had decreased to 12.4% (95% UI: 11.97-12.79); and communicable, maternal, neonatal and nutrition diseases had reduced to 11.8% (95% UI: 10.3 to 12.5).

Considering the total numbers of deaths for all ages, there was an increase of 49% in the absolute number over the period, while deaths due to NCDs increased by 89.7% and corresponded to 1.029 million deaths in 2015. As this is proportional mortality, this increase also reflects population growth and changes in age structure.

The age-standardized death rates for all NCDs for the total population displayed a reduction from 818.6/100,000 (1990) (95% UI: 803.9-834.7) to 611.3/100,000 (2015) (95% UI: 589.8-633.9), representing a reduction of 25.3% between 1990 and 2015 (Table 1).

**Table 1:**
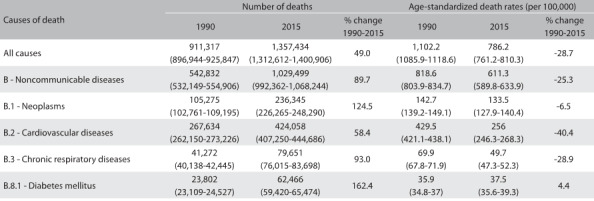
Age-standardized mortality rates for all causes of death and noncommunicable diseases (NCDs) and percentage changes, for both sexes and all ages; Brazil, from 1990 to 2015

Regarding the four groups of NCDs prioritized in the Brazilian and global NCD plans, cardiovascular diseases accounted for 424,058 deaths in 2015, with a rate reduction of 40.5% from 429.5/100,000 inhabitants (1990) (95% UI: 421.1-438.1) to 256/100,000 (2015) (95% UI: 246.3-268.3). Neoplasms accounted for 236,345 deaths in 2015, and the rates went from 142.7/100,000 inhabitants (1990) (95% UI: 139.2-149.1) to 133.5/100,000 (2015) (95% UI: 127.9-140.4), i.e. they remained stable over the period. Chronic respiratory diseases accounted for 79,651 deaths in 2015, with a rate reduction of 28.9% from 69.9/100,000 inhabitants (1990) (95% UI: 67.8-71.9) to 49.7/100,000 (2015) (95% UI: 47.3-52.3). Diabetes mellitus accounted for 62,466 deaths in 2015, and the rates went from 35.9/100,000 inhabitants (1990) (UI: 34.8-37) to 37.5/100,000 (2015) (UI: 35.6-39.3), i.e. they remained stable over the period (Table 1).

At state level, cardiovascular diseases predominated in all 27 federal states and there were reductions in rates between 1990 and 2015, ranging from 16.4% in Tocantins to 47.9% in Rio de Janeiro. The highest rate in 2015 was 353.2/100,000 inhabitants in Maranhão. Chronic respiratory diseases declined in most states, with the largest reduction in Minas Gerais (41.2%). The highest rate in 2015 was recorded in Acre: 77.5/100,000 inhabitants. In contrast, diabetes mellitus increased in most states, with Piauí showing the highest change (84.9%). Some states presented reductions in rates, and the most significant reductions were in São Paulo (28.5%) and in the Federal District (27.1%). The highest diabetes rate in 2015 was in Maranhão: 69.6/100,000 inhabitants. The level of neoplasms remained stable in most states and the highest rate in 2015 was in Amazonas: 155.1/100,000 inhabitants. There were reductions in total NCDs in most states between 1990 and 2015. The highest rate was in Paraíba: 697.3 per 100,000 inhabitants (Table 2).

**Table 2:**
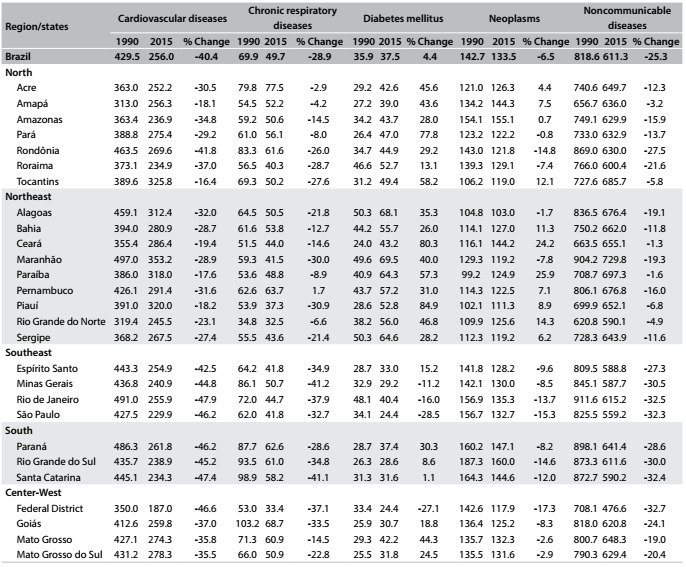
Age-standardized mortality rates for noncommunicable diseases (NCDs) and percentage change according to Brazilian states, for both sexes and all ages, Brazil, from 1990 to 2015

**Figure 2:**
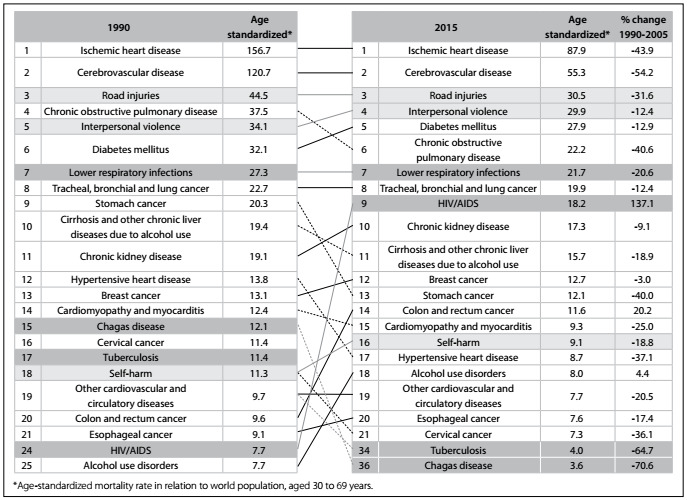
Leading 20 causes of death: age-standardized mortality rate (30 to 69 years of age), rank and percentage change, for both sexes; Brazil, from 1990 to 2015.

In the ranking of the 20 leading causes of premature death according to age-standardized rates (at 30 to 69 years of age) for the years 1990 and 2015, NCDs comprised 15 of them in 2015. At the third level of analysis according to the GBD approach, ischemic heart diseases led throughout the period from 1990 to 2015, with a 43.9% reduction in rates over this period. Cerebrovascular diseases were in second place in the ranking and reduced by 54.2% over the period. Road injury was in third place both in 1990 and in 2015, and interpersonal violence rose from fifth to fourth place over the same period. Diabetes went from sixth to fifth place in the ranking of causes of premature death in 2015, and was followed by chronic obstructive lung diseases (COPD), which fell from fourth to sixth place over the same period. Lower respiratory infections remained in seventh place and lung cancer remained in eighth place over the period. HIV/AIDS increased from 24^th^ place in 1990 to ninth in 2015. Also in 2015, chronic kidney disease was in 10^th^ place, cirrhosis and other chronic liver diseases in 11^th^, breast cancer in 12^th^, stomach cancer in 13^th^, colorectal cancer in 14^th^ (showing growth of 20% in its rate), cardiomyopathy 15^th^, self-harm in 16^th^, hypertensive heart disease in 17^th^, alcohol use disorder in 18^th^, other cardiovascular disease in 19^th^, esophageal cancer in 20^th^ and cervical cancer in 21^st^ position. Also noteworthy were the considerable reductions in tuberculosis from 17^th^ position in 1990 to 34^th^ in 2015 and Chagas disease from 15^th^ to 36^th^ (Figure 2).

## DISCUSSION

It is evident that NCDs were addressed as a problem of health surveillance in Brazil between 1990 and 2015. Based on the results from this study, the burden of noncommunicable diseases has continuously gained in importance, followed by violence, while the biggest improvements came from the sharp reductions in the burden of communicable, maternal, neonatal and nutritional diseases. Age-standardized mortality rates for NCDs presented higher rates throughout the period but with declining trends for cardiovascular diseases and chronic respiratory diseases, while cancer and diabetes showed stable rates between 1990 and 2015 in Brazil.

Regarding premature mortality (at 30 to 69 years of age), NCDs accounted for 15 of the top 20 leading causes. Ischemic heart diseases presented the highest burden, followed by cerebrovascular diseases, which occupied the second place in the ranking. Road injuries were in third place, followed by interpersonal violence, while HIV/AIDS appeared in ninth position.

The data shown here demonstrate the rapid epidemiological transition that Brazil has been undergoing, which has resulted in a high burden of noncommunicable disease.^12^ These changes stem from the underlying demographic transition, with significant decreases in fertility and birth rates, increased life expectancy, an increased proportion of elderly people, rapid urbanization, economic and social growth and unhealthy lifestyles.^7^^,^^12^

Noncommunicable diseases are one of the major public health problems because of their contribution towards high numbers of premature deaths and loss of quality of life. They lead to high degrees of limitations and incapacity to conduct activities of daily living, and also have an economic impact on families, communities and society.^3^^,^^7^^,^^12^^,^^13^

The burden of disease in Brazil shows the presence of increasingly pressing problems such as road injuries and interpersonal violence, which are the third and fourth leading causes of premature death.^14^^,^^15^ Another finding is the dramatic success in reducing the burden of communicable, maternal, neonatal and nutritional diseases. This reduction has been particularly significant in relation to the rapid nutritional transition, which has almost eliminated malnutrition in less than 30 years. However, this transition has also led to a situation in which more than half of the population is overweight and one fifth is obese, consequent to changes in dietary patterns and the sedentary nature of modern life.^12^^,^^16^^,^^17^

The burden of NCDs encompasses individuals in all socioeconomic strata and, more intensely, vulnerable groups such as the elderly and those with low levels of education and income.^1^^,^^3^ Thus, over the last few decades, Brazil has gone from the typical mortality profile of a young population to a picture of more complex and costly diseases that are typical of the more advanced age groups.^12^

NCDs constitute the largest health problem in Brazil.^7^^,^^12^ Estimates for this country have indicated that the losses of labor productivity and decreases in family income resulting from just three NCDs combined (diabetes, heart disease and stroke) contributed a loss of US$ 4.18 billion to the economy between 2006 and 2015.^3^ The direct costs correspond to the expenses involved in medical assistance, medicines, hospitalizations, examinations, procedures, physiotherapy and rehabilitation. The indirect costs are linked to losses in production and income, productivity, jobs and absenteeism. The intangible costs are difficult to estimate and refer to family income, informal care and other matters.^18^

This increase in the burden of NCDs is directly linked to some negative effects from the globalization process, rapid urbanization, sedentary life and high-calorie diets, along with tobacco and alcohol consumption.^1^^,^^7^^,^^13^^,^^19^^,^^20^ These behavioral risk factors have an impact on the major metabolic risk factors, such as overweight/obesity, high blood pressure and increased blood glucose, lipid and cholesterol levels, and these may result in diabetes, cardiovascular disease, stroke and cancer, among other diseases.^1^^,^^12^^,^^19^^,^^20^

Also in Brazil, NCDs predominate as the main cause of mortality, especially cardiovascular diseases. However, the incidence of cardiovascular diseases and consequent mortality have been declining over the last few decades.^12^ Cardiovascular diseases result from metabolic risk factors, such as high blood pressure, inadequate diet, smoking, physical inactivity and other risk factors.^1^^,^^20^ Analysis on mortality trends in Brazil between 2000 and 2011 has shown that, despite an increase in the overall number of deaths due to cardiovascular diseases, the age-adjusted mortality rates for these diseases declined by 24%.^20^ Healthcare delivered by Brazil’s universal public healthcare system, which focuses on primary prevention, has contributed towards this achievement.^20^

Reductions in heart disease mortality rates have varied according to socioeconomic status. A trend analysis on all heart diseases in the city of São Paulo from 1996 to 2010 showed that the decline in the risk of death presented a variable gradient: faster for people living in the wealthiest area and slower for people living in the more deprived neighborhoods.^21^ Stroke mortality trends in Brazil (1979-2009) have shown declines for all stroke subtypes.^22^ The risk of death due to stroke is decreasing in all regions, but the fastest decline in mortality rates is in the wealthiest area, thus contributing to a situation of increasing inequality.^23^

Cancer is already the second highest leading cause of death in most countries and it has been predicted that it will reach first place over the coming years.^1^ In Brazil, over the years studied here, there was stability in the overall rate, but studies have shown distinct trends according to cancer type, age, social status and sex.^15^^,^^24^^,^^25^^,^^26^Among men, there has been an increase in mortality due to prostate and colorectal cancer, and a reduction in lung cancer. Among women, breast, lung and colorectal cancer mortality rates have increased, while cervical and stomach cancer rates have declined.^12^^,^^27^

The incidence of diabetes is growing and it is already among the top ten causes of death worldwide, besides being an important cause of death due to cardiovascular disease. The increase in the death rate due to diabetes has been driven through the growth of the elderly population, the obesity epidemic and unhealthy lifestyles.^28^^,^^29^ The current study showed that the rate for Brazil was stable between 1990 and 2015, although it increased in several states.

Chronic obstructive pulmonary disease (COPD) is the third highest leading cause of death among adults in Brazil. Previous studies in Brazil have shown reductions of mortality due to chronic respiratory diseases,^12^^,^^30^ and these have been attributed to advances in access to primary healthcare and access to medications and reduction of smoking.^12^

The national plan to combat NCDs in Brazil,^19^^,^^31^ WHO’s Global Plan and Sustainable Development Goals have established the target of reducing premature mortality rates or mortality among adults under 70 years of age caused by NCDs.^6^ The evidence from studies shows that Brazil is on track to meet the goal, since the burden arises from diseases that are sensitive to health promotion interventions and care provision.^12^^,^^17^ This positive finding is directly related to implementation of highly cost-effective interventions through the National Health System (Sistema Único de Saúde, SUS), such as expansion of primary care and wide distribution of drugs to the population that is at high risk of developing cardiovascular diseases, as well as measures established to control tobacco use.^12^^,^^17^^,^^20^

This study also draws attention to the importance of external causes of death and the magnitude of such occurrences, caused by interpersonal violence and traffic accidents. These events are particularly frequent among young people and contribute towards premature mortality during the productive phase.^14^ Also noteworthy are premature deaths due to HIV, and the rise of HIV in the ranking of causes of premature mortality over the last few decades.

It is important to emphasize the need to ensure quality in mortality estimates in Brazil. Problems regarding the quality of information about deaths have arisen in the past. Thus, analysis based on the GBD study is of great use, particularly for mortality assessment, since it uses methods for correction of underreporting of deaths and for redistribution of garbage codes (deaths attributed to causes that may not be the real causes of death or are not properly described). However, any process of data correction needs to be regarded with caution, since assumptions made in using these methods may lead to overestimation or underestimation of the degree of coverage, thereby generating bias in the estimates of mortality coverage.^32^^,^^33^There is still a long way to go to reduce these causes of death and illness. The diseases described here have a long course and require a comprehensive longitudinal approach, with dedicated investment in self-care and bonding.^34^ It is therefore essential to reduce inequities within healthcare, so as to ensure access to care for the entire population, especially the most vulnerable groups, given the higher concentration of NCDs and their risk factors in the low-income and low-education population.^1^^,^^3^

SUS and universal access to healthcare in Brazil act in a protective manner, especially with regard to care for the great majority of the population. It is important to highlight the role of SUS in achieving the results regarding reduction in mortality due to NCDs that are presented here.^12^^,^^17^^,^^20^

## CONCLUSION

This study shows the epidemiological transition in Brazil between 1990 and 2015, with increasing proportional mortality due to NCDs, followed by violence, and decreasing mortality due to communicable, maternal and neonatal causes within the global burden of diseases. NCDs had the highest mortality rates over the whole period, but with reductions in cardiovascular diseases, chronic respiratory diseases and cancer. Diabetes increased over this period. NCDs were the leading causes of premature deaths (30 to 69 years of age).
